# Dental floss traction-assisted precut sphincterotomy for difficult biliary cannulation in elongated papilla

**DOI:** 10.1055/a-2615-5848

**Published:** 2025-07-04

**Authors:** Ping Wang, Wenguang Yang, Yuhong Ren, Bin Yang, Sichao Wen, Haiyong Long, Mingwen Guo

**Affiliations:** 1 Department of Gastroenterology, Qionglai Medical Center Hospital, Qionglai, China

Endoscopic retrograde cholangiopancreatography (ERCP) is a cornerstone intervention for biliary and pancreatic diseases, achieving >95% cannulation success. However, challenging cases persist. The European Society of Gastrointestinal Endoscopy (ESGE) defines difficult cannulation as procedures requiring >5 attempts, lasting >5 minutes, or resulting in unintended pancreatic duct cannulation 1 2 . Anatomical variations (e.g., elongated/Shar-Pei-like papillae) and pathological changes (strictures and tumors) are key contributors 3 4 .

A 78-year-old man with pancreatitis underwent computed tomography (CT)/magnetic resonance imaging (MRI)/endoscopic ultrasonography (EUS) revealing pancreatic duct dilation and a distal stone ( Fig. 1 **a** , Video 1 ). Due to a large pancreatic duct stone, ERCP with pancreatic duct stent insertion was initially performed, followed by extracorporeal shock wave lithotripsy and stone extraction. During ERCP, the major duodenal papilla was identified as elongated and Shar-Pei-like, with its orifice obscured by overlying mucosal folds ( Fig. 1 **b** ), resulting in failed pancreatic duct cannulation. Thus, we adopted dental floss traction to expose the papilla: preassembled ex vivo with a titanium clip (Harmony Clip; Micro-Tech Co., Ltd., Nanjing, China) and dental floss ( Fig. 1 **c** ), selecting the mucosa above the papillary orifice as the traction point ( Video 1 ). After releasing the titanium clip, traction on the floss effectively exposed the papilla ( Fig. 1 **d** ). Due to the small, soft papillary opening, cannulation still failed. After the papilla was stabilized by dental floss traction with a titanium clip, the improved fixation facilitated precise precut sphincterotomy. We then used an Olympus DualKnife (KD-311; Olympus Medical, Tokyo, Japan) for minor pre-cutting ( Fig. 1 **e** ). Selective pancreatic duct cannulation was successfully achieved, with the stent placed after guide wire advancement into the body/tail of the pancreatic duct ( Fig. 1 **f** ). For large, long, and soft duodenal papillae, floss traction not only optimally exposed the papillary opening but also stabilized the papilla for subsequent procedures like pre-cutting, thereby enhancing ERCP cannulation success.

**Fig. 1:**
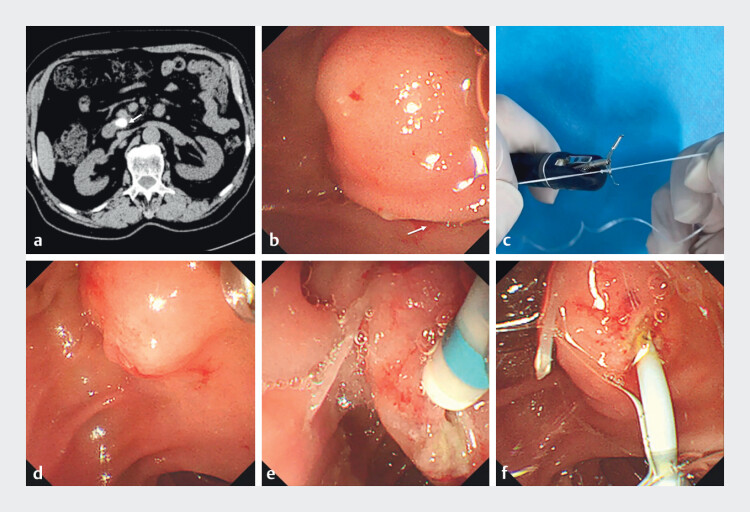
Dental floss traction-assisted precut sphincterotomy for difficult biliary cannulation in elongated papilla. **a** Computed tomography showed pancreatic duct dilation with a stone in the distal pancreatic duct. **b** Endoscopic view of the major duodenal papilla, demonstrating an elongated and Shar-Pei-like morphology with the orifice obscured by overlying mucosal folds. **c** Preparation of the dental floss traction device: preassembled ex vivo with a titanium clip and dental floss. **d** Traction on the floss effectively exposed the papilla after releasing the titanium clip. **e** Minor pre-cutting was then carried out using an Olympus DualKnife. **f** Final placement of the pancreatic duct stent confirmed by fluoroscopy.

This video demonstrates dental floss traction-assisted precut sphincterotomy for successful pancreatic duct cannulation in a case of difficult biliary access due to an elongated papilla.Video 1

Endoscopy_UCTN_Code_TTT_1AR_2AC

## Notes

Endoscopy E-Videos https://eref.thieme.de/e-videos
*E-Videos* is an open access online section of the journal *Endoscopy* , reporting on interesting cases and new techniques in gastroenterological endoscopy. All papers include a high-quality video and are published with a Creative Commons CC-BY license. Endoscopy E-Videos qualify for HINARI discounts and waivers and eligibility is automatically checked during the submission process. We grant 100% waivers to articles whose corresponding authors are based in Group A countries and 50% waivers to those who are based in Group B countries as classified by Research4Life (see: https://www.research4life.org/access/eligibility/ ). This section has its own submission website at https://mc.manuscriptcentral.com/e-videos .
